# Genomewide Association Study of Statin‐Induced Myopathy in Patients Recruited Using the UK Clinical Practice Research Datalink

**DOI:** 10.1002/cpt.1557

**Published:** 2019-07-31

**Authors:** Daniel F. Carr, Ben Francis, Andrea L. Jorgensen, Eunice Zhang, Hector Chinoy, Susan R. Heckbert, Joshua C. Bis, Jennifer A. Brody, James S. Floyd, Bruce M. Psaty, Mariam Molokhia, Maryse Lapeyre‐Mestre, Anita Conforti, Ana Alfirevic, Tjeerd van Staa, Munir Pirmohamed

**Affiliations:** ^1^ Wolfson Centre for Personalised Medicine Department of Molecular and Clinical Pharmacology Institute of Translational Medicine University of Liverpool Liverpool UK; ^2^ Department of Biostatistics Institute of Translational Medicine University of Liverpool Liverpool UK; ^3^ Rheumatology Department Salford Royal NHS Foundation Trust Manchester Academic Health Science Centre Salford UK; ^4^ NIHR Manchester Biomedical Research Centre Manchester University NHS Foundation Trust Manchester Academic Health Science Centre The University of Manchester Manchester UK; ^5^ Cardiovascular Health Research Unit Department of Epidemiology University of Washington Seattle Washington USA; ^6^ Cardiovascular Health Research Unit Department of Medicine University of Washington Seattle Washington USA; ^7^ Kaiser Permanente Washington Health Research Institute Seattle Washington USA; ^8^ School of Population Health and Environmental Sciences King's College London London UK; ^9^ Medical and Clinical Pharmacology CHU Toulouse Toulouse France; ^10^ University Hospital Verona Italy; ^11^ Health e‐Research Centre School of Health Sciences Faculty of Biology, Medicine, and Health University of Manchester Manchester UK; ^12^ Faculty of Science Division of Pharmacoepidemiology and Clinical Pharmacology Utrecht University Utrecht The Netherlands

## Abstract

Statins can be associated with myopathy. We have undertaken a genomewide association study (GWAS) to discover and validate genetic risk factors for statin‐induced myopathy in a “real‐world” setting. One hundred thirty‐five patients with statin myopathy recruited via the UK Clinical Practice Research Datalink were genotyped using the Illumina OmniExpress Exome version 1.0 Bead Chip and compared with the Wellcome Trust Case‐Control Consortium (*n* = 2,501). Nominally statistically significant single nucleotide polymorphism (SNP) signals in the GWAS (*P* < 5 × 10^−5^) were further evaluated in several independent cohorts (comprising 332 cases and 449 drug‐tolerant controls). Only one (rs4149056/c.521C>T in the *SLCO1B1* gene) SNP was genomewide significant in the severe myopathy (creatine kinase > 10 × upper limit of normal or rhabdomyolysis) group (*P* = 2.55 × 10^−9^; odds ratio 5.15; 95% confidence interval 3.13–8.45). The association with *SLCO1B1* was present for several statins and replicated in the independent validation cohorts. The data highlight the role of *SLCO1B1* c.521C>T SNP as a replicable genetic risk factor for statin myopathy. No other novel genetic risk factors with a similar effect size were identified.

**Study Highlights**

WHAT IS THE CURRENT KNOWLEDGE ON THE TOPIC?

☑ Risk of statin‐induced myopathy is associated with variation of the *SLCO1B1* gene, which encodes the OATP1B1 hepatic uptake transporter, of which statins are substrates. To date, no other validated genetic risk factors have been identified.

WHAT QUESTION DID THIS STUDY ADDRESS?

☑ Undertaking a genomewide association study in a “real‐world” patient cohort recruited via the Clinical Practice Research Datalink, this study aimed to determine whether any other novel genetic risk loci for statin myopathy could be identified.

WHAT DOES THIS STUDY ADD TO OUR KNOWLEDGE?

☑ The study suggests that, aside from SLCO1B1, no other risk loci for statin myopathy are apparent. The unexplained statin myopathy risk is likely due to nongenetic risk factors or the influence of rare genetic variants analyzed in this study.

HOW MIGHT THIS CHANGE CLINICAL PHARMACOLOGY OR TRANSLATIONAL SCIENCE?

☑ Common genetic variants do not seem to explain statin myopathy risk. The data presented seem to suggest that future translational work in this field should focus on rare variant analysis and on identifying nongenetic risk factors.

The 3‐hydroxy‐3‐methyl‐glutaryl‐coenzyme A inhibitors, or statins, are a widely prescribed class of drugs for the treatment of hyperlipidemia. Although generally well tolerated, a small proportion of patients can develop muscle‐related adverse effects.^1^ These can range from mild muscle pain without creatine kinase (CK) elevation, where causality can be difficult to assess, to myopathy where the CK becomes elevated (>4 × upper limit of normal (ULN)), with the most extreme reactions being rhabdomyolysis with renal impairment.^2^ A systematic review suggested that the incidence of statin‐induced mild muscle pain is 190 cases/100,000 patient years with myopathy and rhabdomyolysis at 5 and 1.6 cases/100,000 patient years, respectively.^3^

A number of genetic studies^4^, ^5^, ^6^, ^7^, ^8^ have identified a nonsynonymous polymorphism (p.V147L/c.521C>T) in the *SLCO1B1* gene (rs4149056), encoding a hepatic uptake transporter protein as a predisposing factor for statin myopathy. Our pilot proof‐of‐principle candidate gene study, which analyzed a subset of the cohort in this study (77 cases and 372 statin‐tolerant controls) replicated the association between the *SLC01B1* gene polymorphism and statin myopathy^9^ showing the validity of our recruitment strategy via the UK Clinical Practice Research Datalink (CPRD), an electronic health record database. The association with the *SLCO1B1* gene has biological plausibility in that it leads to impaired hepatic uptake of statins by the transporter,^10^, ^11^ causing increased circulating drug concentrations.^12^, ^13^ However, to date, no other clinically relevant, reproducible genetic variants have been identified.

Other genetic markers have been associated with statin‐myopathy, including polymorphisms in the coenzyme Q2 4‐hydroxybenzoate polyprenyltransferase (*COQ2*)^14^ and human eyes shut ortholog^15^ genes, but have not been independently replicated. The genetic association of statin myopathy with the *GATM* gene^16^ has also not been replicated.^17^, ^18^

Utilizing patients with statin myopathy recruited via CPRD,^19^ the aims of our study were twofold: to undertake a genomewide association study (GWAS) to identify novel genetic risk factors predisposing individuals to statin‐induced myopathy, and to validate any association in independent patient groups and perform meta‐analysis of any association signals. Taking all cohorts together, this represents the largest GWAS of the pharmacogenetics of statin myopathy undertaken to date.

## RESULTS

### Case‐control discovery GWAS

A total of 128 of 135 myopathy case samples and 654,642 single nucleotide polymorphisms (SNPs) passed the predefined genotyping quality control (QC) criteria. Of the seven individuals excluded, three failed sample call‐rate criteria, three failed the gender identity check (due to sample mislabeling), and one individual was excluded as a population outlier after principal component analysis (**Figure S1**). The individual statins responsible for the muscle toxicity are shown in **Table S1**.

**Figure 1:**
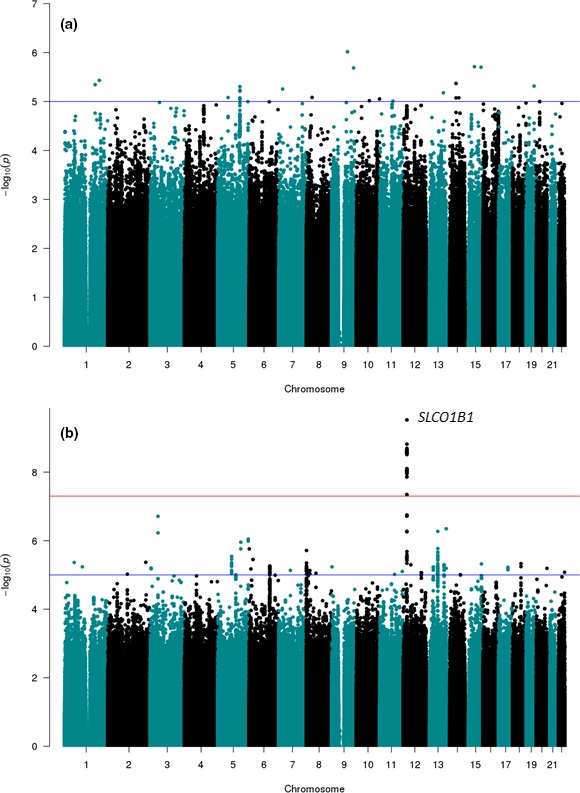
Manhattan plot of genomewide association analysis of statin‐induced myopathy. The data represents logistic regression derived log *P* values (*y*‐axis) of single nucleotide polymorphisms (SNPs) for the discovery case‐control analysis of (**a**) the “all myopathy” phenotype (*n* = 128) and (**b**) the “severe myopathy” sub‐phenotype (*n* = 32) with the Wellcome Trust Case‐Control Consortium 2 (WTCCC2) population controls (*n* = 2,501). *X*‐axis is the position of the SNP with the chromosome indicated. [Colour figure can be viewed at http://www.wileyonlinelibrary.com]

From the case‐control discovery GWAS, a total of 21 SNPs were initially identified as notionally significant (*P* < 5 × 10^−5^; 12 from the all myopathy analysis and 9 from the severe myopathy analysis; **Figure** 1 **a,b**, respectively). However, only one signal reached genomewide significance: rs4149056 in the *SLCO1B1* locus (*P* = 2.5 × 10^−9^; **Table** 1). Sensitivity analysis of discovery cases (all myopathy) for simvastatin cases only (**Figure S3** and **Table S4**) showed no genomewide significant association signals (*P* > 5 × 10^−8^), although *SLCO1B1* was among the top associated loci. Similarly, sensitivity analysis of atorvastatin cases only (**Figure S4** and **Table S5**) also showed no genomewide significant associations. *SLCO1B1* was not identified in the top associated loci.

**Table 1:** SNPs suggested to be associated with all statin‐induced myopathy and severe myopathy from the discovery case‐control analysis, replication analysis, independent simvastatin and cerivastatin study analyses, and the combined meta‐analysis

All myopathy			Discovery case‐control study CPRD cases (*n* = 128) vs. WTCCC (*n* = 2,501)		Replication study EUDRAGENE cases (*n* = 19) vs. CPRD statin‐tolerant (*n* = 585)		Simvastatin validation case‐control study definite/incipient myopathy cases (*n* = 141) vs. controls (*n* = 4,046)		Cerivastatin validation case‐control study cases (*n* = 172) vs. controls (*n* = 361)		Combined meta‐analysis cases (*n* = 460) vs. controls (*n* = 7,493)		
rs#	Chr	Gene	Per allele OR (95% CI)	*P* value	Per allele OR (95% CI)	*P* value	Per allele OR (95% CI)	*P* value	Per allele OR (95% CI)	*P* value	Per allele OR (95% CI)	*P* value	*I* ^2^
rs36121096	5	*PDE4D*	3.82 (2.20–6.62)	2.0 × 10^−5^	0 (0–∞)	1.00	1.37 (0.56–6.07)	0.61	1.51 (0.60–3.82)	0.38	2.01 (1.22–3.31)	0.006	0.00
rs55902659	5	*SLC12A2*	0.44 (0.31–0.66)	4.9 × 10^−6^	0.42 (0.15–1.18)	0.10	0.81 (0.76–1.90)	0.24	1.00 (0.72–1.37)	0.99	0.74 (0.59–0.92)	0.008	0.84
rs17359612	9	*TLE1*	2.49 (1.71–3.64)	1.1 × 10^−5^	1.59 (0.58–4.35)	0.36	1.25 (0.51–4.17)	0.54	1.21 (0.63–2.33)	0.56	1.67 (1.19–2.34)	0.003	0.00
rs79860430	14	*ATG14*	2.59 (1.76–3.82)	8.4 × 10^−6^	0 (0–∞)	1.00	1.13 (0.34–8.16)	0.81	1.27 (0.62–2.58)	0.51	2.17 (1.48–3.17)	7.61 × 10^‐5^	0.53
rs77855582	16	*GALNS*	3.88 (2.25–6.69)	1.9 × 10^−5^	3.60 (0.99–13.0)	0.05	1.61 (0.78–4.55)	0.45	NA	NA	NA	NA	NA

Severe myopathy			Discovery case‐control study CPRD cases (*n* = 32) vs. WTCCC (*n* = 2,501)		Replication study EUDRAGENE cases (*n* = 13) vs. CPRD statin‐tolerant (*n* = 585)		Simvastatin validation case‐control study definite myopathy cases (*n* = 54) vs. controls (*n* = 4,046)		Cerivastatin validation case‐control study cases (*n* = 172) vs. controls (*n* = 361)		Combined meta‐analysis cases (*n* = 271) vs. controls (*n* = 7,493)		
rs#	Chr	Gene	Per allele OR (95% CI)	*P* value	Per allele OR (95% CI)	*P* value	Per allele OR (95% CI)	*P* value	Per allele OR (95% CI)	*P* value	Per allele OR (95% CI)	*P* value	*I* ^2^
rs73089338	3	*CDCP1*	4.63 (2.70–7.96)	1.9 × 10^−7^	1.07 (0.25–4.54)	0.92	0.70 (0.26–1.90)	0.49	1.02 (0.49–2.16)	0.95	1.94 (1.27–2.98)	0.002	0.86
rs504365	5	*RASGRF2*	0.18 (0.07–0.44)	2.9 × 10^−6^	1.63 (0.73–3.65)	0.23	1.60 (0.99–2.57)	0.06	1.28 (0.94–1.75)	0.11	1.17 (0.92–1.50)	0.20	0.82
rs2247256	8	*ERICH1*	0.16 (0.06–0.43)	1.9 × 10^−6^	1.81 (0.80–4.07)	0.15	0.58 (0.36–0.91)	0.02	1.03 (0.76–1.40)	0.85	0.78 (0.61–0.99)	0.04	0.82
rs117576073	11	*CYP2R1*	8.36 (3.66–19.06)	2.8 × 10^−5^	0 (0–∞)	1.00	0.48 (0.03–8.68)	0.62	0.31 (0.04–2.31)	0.26	3.11 (1.25–7.78)	0.01	0.79
rs4149056	12	*SLCO1B1*	5.15 (3.13–8.45)	**2.5** × **10** ^**−9**^	3.98 (1.75–9.03)	0.001	4.91 (3.09–7.77)	**1.3** × **10** ^−**11**^	1.86 (1.32–2.62)	3.9 × 10^−4^	2.99 (2.34–3.82)	**2.63** × **10** ^−**18**^	0.87
rs4149000	12	*SLCO1A2*	3.94 (2.36–6.57)	2.9 × 10^−6^	2.53 (1.00‐6.39)	0.050	7.29 (4.13–12.8)	**6.5** × **10** ^−**12**^	1.74 (1.16–2.60)	0.007	2.81 (2.10–3.75)	**3.31** × **10** ^−**12**^	0.91
rs28447350	13	Intergenic	3.66 (2.23–6.00)	5.3 × 10^−7^	0.55 (0.21–1.43)	0.219	0.88 (0.49–1.59)	0.676	1.06 (0.73–1.52)	0.76	1.32 (1.01–1.74)	0.04	0.84

Data indicates *P* values and ORs (95% CI; per‐allele) derived from logistic regression for discovery cohort vs. WTCCC cohort (*n* = 2,501). Only associations < 5 × 10^−5^ in the initial discovery cohort are shown with those reaching genomewide significance (*P* < 5 × 10^−8^) highlighted in bold.

CI, confidence interval; CPRD, Clinical Practice Research Datalink; NA, not available; OR, odds ratio; SNPs, single nucleotide polymorphisms; WTCCC, Wellcome Trust Case‐Control Consortium.

Univariate analysis of nongenetic variables for myopathy (*n* = 128) and severe myopathy (*n* = 32) cases vs. statin‐tolerant controls (*n* = 585) was undertaken (**Table S1**). Age, gender, body mass index, antihypertensive comedication, occurrence of cramps, and history of hypertension showed an association with *P* < 0.10 for all myopathy and mean daily dose, age, and occurrence of cramps showed an association with *P* < 0.10 for “severe myopathy.” These variables were incorporated into the replication cohort logistic regression model for case control analysis.

### Replication cohort analysis

Twenty‐one SNPs below a threshold of *P* < 5 × 10^−5^ in the discovery GWAS analysis were carried forward for genotyping in the “Replication Cohort” (consisting of the CPRD statin‐exposed controls (*n* = 585) and the EUDRAGENE statin‐myopathy cases (*n* = 19)). A total of 9 SNPs (two from the severe myopathy analysis and seven from the all myopathy analysis), however, were subsequently excluded because of inability to design TaqMan or MassArray assays due to proximal sequence constraints (*n* = 4), low genotyping call rate (*n* = 4), and Hardy−Weinberg deviation (*n* = 1). Thus, a total of 12 SNPs were genotyped (5 for all myopathy and 7 for severe myopathy; **Table** 1).

Data from simvastatin and atorvastatin cases only sensitivity analyses for the 12 SNPs initially identified in the overall discovery cohort is reported in **Table S3**. None of the 12 signals showed a statistically significant association with atorvastatin myopathy. Eight loci were notionally associated with simvastatin “all myopathy” albeit not to genomewide significance.

#### Replication cohort

Candidate gene analysis of the 19 EUDRAGENE myopathy cases (13 severe) with the 585 statin‐tolerant controls identified 2 of 12 associations of nominal significance (*P* < 0.05), both of which were for the severe myopathy phenotype (**Table** 1). These were *SLCO1B1* rs4149056 (*P* = 0.001; odds ratio (OR) 3.98; 95% confidence interval (CI) 1.75–9.03) and *SLCO1A2* rs4149000 (*P* = 0.05; OR 2.53; 95% CI 1.00–6.39). No other statistically significant associations were observed in the other nine SNPs (*P* < 0.05). Minor allele frequencies for the 12 SNPs were comparable for both simvastatin and atorvastatin controls.

### Association signal validation

#### “Simvastatin” cohort

Summary statistics from this cohort (**Table** 1) showed a genomewide significant association for 2 of 12 SNPs, both of which were associated with the severe myopathy phenotype in the initial analysis. Both rs4149056 in *SLCO1B1* and rs4149000 in *SLCO1A2* were significantly associated with both definite myopathy (*P* = 7.30 × 10^−14^ and *P* = 7.62 × 10^−11^, respectively) and the incipient or definite myopathy phenotype (*P* = 1.33 × 10^−11^ and 6.49 × 10^−12^). None of the other SNPs were significantly associated with either myopathy phenotype (*P* > 0.05).

#### “Cerivastatin” cohort

Summary statistics from the cerivastatin rhabdomyolysis validation cohort (**Table** 1) showed that the same two SNPs (rs4149056 and rs4149000) showed a significant (albeit not to a genomewide threshold) association (3.90 × 10^−4^ and 0.007, respectively). None of the other SNPs showed an association (*P* > 0.05).

### Further analysis of the SLCO1B1 and SLCO1A2 signals

The two SNPs that seem to be strongly associated with statin myopathy in the discovery and replication cohorts are within two gene loci (*SLCO1B1* and *SLCO1A2*) that are in a strong block of linkage disequilibrium in the discovery cohort (data not shown). Conditional analysis correcting for the *SLCO1B1* rs4149056 genotype was undertaken. The analysis of the 32 discovery severe myopathy cases vs. 585 statin‐tolerant controls abolished the rs4149000 genotype association (*P* = 0.934), as well as for the all myopathy phenotype (*P* = 0.368) indicating that the two risk alleles are not acting in *cis* on the same haplotype and that the *SLCO1A2* association is not acting independently of *SLCO1B1*.

### Meta‐analysis of SLCO1B1 and SLCO1A2 association signals

**Figure 2:**
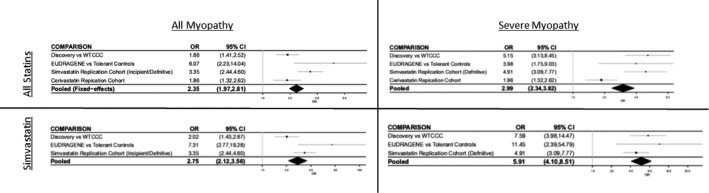
Forest plot depicting meta‐analysis for the *SLCO1B1* c.521T>C (rs4149056) polymorphism for both “all myopathy” (creatine kinase (CK) > 4 × upper limit of normal (ULN)) and “severe myopathy” (CK > 10 × ULN/rhabdomyolysis) phenotypes caused by all statins (upper panels) and simvastatin only (lower panels).  CI, confidence interval; OR, odds ratio;  WTCCC, Wellcome Trust Case‐Control Consortium.

Meta‐analysis combining the discovery and all replication cohorts (limited to severe cases only; 271 cases vs. 7,493 controls) yielded a meta‐analytic genomewide significant *P* value for SLCO1B1, rs4149056 (*P* = 2.63 × 10^−18^; OR 2.99; 95% CI 2.34–3.82; **Table** 1), highlighting the predominant role of *SLCO1B1* in predisposing to myopathy caused by a variety of statins. An increased statistical significance of the association signal within the *SLCO1A2* was also observed, but no other meta‐analysis demonstrated an increased statistical significance of the signal initially identified in the discovery case‐control study (**Table** 1). Meta‐analysis of the *SLCO1B1* signal for rs4149056 limited to the simvastatin‐exposed cases and controls only (**Figure** 2) led to a *P* value of 1.46 × 10^−21^ (OR 5.91; 95% CI 4.10–8.51; *I* ^2^ = 1.00) for the severe myopathy phenotype and a *P* value of 2.01 × 10^−14^ (OR 2.75; 95% CI 2.12–3.56; *I* ^2^ = 0.78) for the all myopathy phenotype.

## DISCUSSION

Our discovery GWAS identified 12 SNPs, which were nominally associated with either all myopathy (CK > 4 × ULN ± muscle symptoms) or severe myopathy (CK > 10 × ULN or rhabdomyolysis). Replication was undertaken in three separate patient cohorts, which showed that only the previously identified^6^, ^9^ and widely replicated c.521C>T variant (rs4149056) in *SLCO1B1* and an intronic SNP in the *SLCO1A2* gene were risk factors for statin myopathy. The latter, however, was not significant after adjustment for *SLCO1B1* genotype. Our data concur with a previous statin myopathy GWAS,^9^ as well as our own pilot data^9^ and other candidate gene studies,^4^, ^5^, ^8^, ^20^ that the *SLCO1B1* c.521C>T polymorphism (rs4149056) is the predominant genetic risk factor for statin‐induced myopathy. Our finding is also consistent with a recent meta‐analysis of 14 studies comprising 3,265 myopathy patients and 7,743 controls.^21^ Additionally, previously reported associations in the *GATM*,^16^ *COQ2*,^14^ and human eyes shut ortholog^15^ gene loci were not replicated.

Our discovery cohort was heterogeneous in terms of the severity of myopathy and statin implicated. The association with the rs4149056 variant in *SLCO1B1* was stronger in patients with the severe form of myopathy (CK > 10 × ULN or rhabdomyolysis) irrespective of the statin involved reaching genomewide significance (**Figure** 2). The lower effect size observed in patients with the less severe form of statin myopathy (defined in our discovery cohort as CK > 4 × ULN) may reflect multiple causes in the mildly affected cases and the difficulty in attributing causality to statins in all cases.

Of the different statins implicated, simvastatin was the most common, accounting for 66% of our cases and 69% of the severe cases. Meta‐analysis of our discovery severe myopathy cohort with the simvastatin definite myopathy cohort did strengthen the association (*P* = 7.17 × 10^−19^) with little evidence of heterogeneity between the two (*I* ^2^ = 1). Further incorporation of the cerivastatin cohort marginally weakened the association in keeping with the different effect sizes of the *SLCO1B1* locus for different statins. These data are consistent with the fact that the pharmacokinetic effect of the *SLCO1B1* variant is greatest for simvastatin. The area under the curve for simvastatin acid is increased by 221% in CC homozygotes compared with those individuals who are TT homozygotes for the *SLCO1B1* c.521C>T polymorphism.^13^ Corresponding values for the other statins are as follows: atorvastatin (145%),^22^ fluvastatin (19%),^23^ lovastatin acid (186%),^24^ pitavastatin (208%),^25^ pravastatin (91%),^23^ and rosuvastatin (65%).^22^ No similar data are available for cerivastatin. Based on these pharmacokinetic data and the results of our data, together with the recent meta‐analysis,^21^ it might be suggested that *SLCO1B1* locus is important for all statins, but the effect size will likely vary, being greatest for simvastatin and lowest for fluvastatin.

The aim of our GWAS was to identify other loci associated with statin myopathy. Apart from the association with *SLCO1B1*, we also identified an association with an SNP located in the 5′UTR of the *SLCO1A2* locus (**Figure S2**). This signal, however, was not independent of the *SLCO1B1* signal. *SLCO1A2* encodes SLCO1A2, a hepatic‐expressed efflux transporter,^26^ which is responsible for the sodium‐independent transport of organic anions, such as bromosulfophthalein, taurocholate, and unconjugated cholate bile acids.^27^, ^28^ SLCO1A2 has also been shown to have substrate specificity for pitavastatin^29^ and rosuvastatin,^30^ but, to date, there is no evidence of its ability to transport simvastatin or atorvastatin. Determination of whether the *SLCO1A2* locus can act as an independent risk factor for statin myopathy will require a much larger sample size. None of the other loci identified in the discovery cohort were replicated in any of the cohorts, and meta‐analysis did not provide any indication that these loci acted as predisposing factors for statin myopathy.

For a number of years, the c.521T>C *SLCO1B*1 variant has been recognized as a clinically important risk factor for statin‐induced myopathy, particularly with regard to simvastatin, and to a lesser extent atorvastatin. Indeed, summary of product characteristics labeling for both drugs^31^, ^32^ highlights the increased risk of myopathy in individuals who are carriers of the low‐activity C allele. It has been suggested that the maximum dose of simvastatin, pitavastatin, and atorvastatin should be reduced by fourfold in individuals who are CC homozygotes based on pharmacokinetic calculations.^33^ Interestingly, a recent small randomized trial (*n* = 159) of patients not on statins because of prior myalgia attributed to a statin showed that providing information on the *SLCO1B1* genotype improved statin reinitiation and low‐density lipoprotein‐cholesterol lowering, but not adherence, when compared with the usual care arm.^34^

It is clear that although S*LCO1B1* is a key risk factor for statin myopathy, it does not explain a significant proportion of the interindividual variability in statin toxicity. Genetic studies to date have been limited by recruitment of significant numbers of cases of what is a rare adverse drug reaction. As such, many studies lack statistical power to detect small effect sizes and have, in fact, only identified the “low hanging fruit.” It is possible that much of the heritability of statin myopathy risk may lie either with rare variants of large effect sizes or with other common genetic loci with small to modest effect sizes, which will require much larger patient numbers. A major issue is that we do not fully understand the mechanism of statin myopathy and muscle damage, apart from the fact that high doses or high statin concentrations increase risk. Further functional studies to uncover the mechanisms of muscle damage induced by statins will be important in elucidating further predisposing factors.

In conclusion, our data further confirm the predominant role of the *SLCO1B1* c.521 C>T polymorphism in predisposing to statin‐induced myopathy in a “real‐world” patient population, in particular with simvastatin. Moreover, the data failed to identify other statin myopathy‐associated genetic risk factors. However, this meta‐analysis is relatively small and may lack statistical power. The additional signal identified in the *SLCO1A2* locus was not independent of *SLCO1B1*, but may require further investigation from a functional perspective to determine the role of this transporter in statin transport.

## METHODS

**Figure 3:**
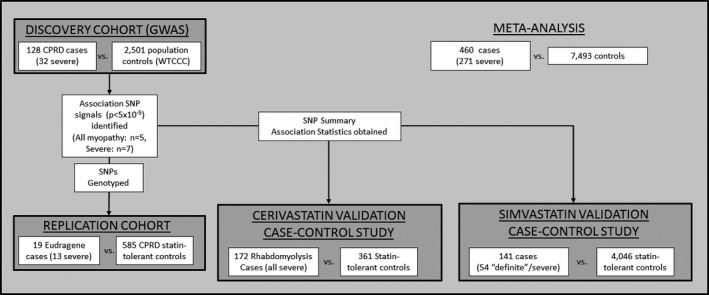
Schematic representation of the discovery, replication, and validation cohort case‐control analyses and subsequent meta‐analysis performed. Patient numbers represent those included in analyses post‐sample quality control. CPRD, Clinical Practice Research Datalink; GWAS, genomewide association study; SNP, single nucleotide polymorphism; WTCCC, Wellcome Trust Case‐Control Consortium 2.

The study design is summarized in **Figure** 3. Briefly, a discovery case‐control GWAS was undertaken, followed by candidate variant replication in a second case‐control cohort. The same association signals were validated in existing data from two independent validation case‐control studies.

### CPRD case‐control recruitment

From a cohort of ~ 600,000 patients receiving statins identified in the CPRD (http://www.cprd.com), a case‐control design was used to identify suitable patients for the study, as previously described.^19^, ^35^ Participation was restricted to white people ≥18 years of age and with the first ever statin prescription at least 1 year after the start of CPRD data collection.

All cases conformed to internationally agreed standards for statin‐induced myopathy and rhabdomyolysis.^2^ Cases were categorized into two groups: (i) myopathy: patients who discontinued their implicated statin with a rise in CK > 4 × ULN; and (ii) severe myopathy: individuals with a history of rhabdomyolysis or CK > 10 × ULN after statin exposure. Controls were defined as individuals receiving statins for at least 3 months with no history of abnormal serum CK measurements.

General practitioners (GPs) were contacted with a list of potential cases and/or controls identified from their practice. They were first asked to review the medical records of listed individuals and remove any patients they considered to not fulfill the case or control criteria. They were then asked to contact suitable patients by letter requesting participation. Individuals who gave written informed consent were invited to provide either a saliva sample (by post) or a blood sample (by visiting the practice). All samples were then forwarded on to the University of Liverpool for processing. To preserve anonymity, patient and practice identifier codes were used throughout the recruitment process and all patient contact was via the GP only. A total of 149 myopathy cases and 585 controls were recruited between April 2010 and June 2013, although only 135 cases were available at the time of genotyping.^19^ Relevant clinical and demographic data (summarized in **Table** 1) was retrospectively obtained from the CPRD.

In addition to the above, five supplemental cases of statin‐induced myopathy conforming to our phenotype criteria were identified in the tertiary adult muscle clinic run through Salford Royal NHS Foundation Trust, UK, and recruited into the UKMYONET genetic study.^36^

### Additional cohorts and studies

Three replication cohorts were utilized to validate associations identified in the case‐control discovery GWAS (summarized in **Figure** 3).

#### EUDRAGENE cohort

A total of 19 adults (>18 years of age) with statin‐exposed myopathy (5 simvastatin, 5 atorvastatin, 2 rosuvastatin, and 1 fluvastatin) matching the case phenotype (defined above) were recruited using spontaneous adverse drug reaction reports and laboratory CK results from the European pharmacovigilance center and UK primary care practices from 2006−2012 via the EUDRAGENE collaborative network.^37^ All cases were adjudicated using internationally accepted criteria for myopathy and severe myopathy^2^ by an independent panel, consisting of clinicians and pharmacovigilance experts. Of the 19 cases, 13 were categorized as having severe myopathy. EUDRAGENE cases were combined with the 585 CPRD statin‐tolerant controls to form the “Replication cohort.”

#### The “simvastatin” validation case‐control study

A total of 141 patients with simvastatin myopathy were recruited via the SEARCH collaborative group.^6^ These consisted of 54 “definite” statin myopathies (defined as otherwise unexplained muscle symptoms with CK > 10 × ULN) and 87 “incipient” statin myopathies (defined as alanine aminotransferase > 1.7 × ULN and CK both > 5 × baseline and > 3 × ULN). For the purpose of meta‐analysis, the “definite” myopathy phenotype aligned to these studies was severe myopathy phenotype with both “incipient and definite” aligning to the all myopathy phenotype. A total of 4,046 statin‐tolerant controls were included from the SEARCH and Heart Protection Study groups.^6^, ^38^

#### The “cerivastatin” validation case‐control study

The study sample consisted of data from 172 cases of cerivastatin rhabdomyolysis and 361 statin‐using controls from the Heart and Vascular Health study, as previously reported.^7^ All cases and controls were of European ancestry. Controls with creatine kinase levels > 10 × ULN were excluded.

##### Study approvals

Ethical approval for recruitment via CPRD was obtained from the National Research Ethics Committee North West 2 – Liverpool Central. Furthermore, approval to use the CPRD data was obtained from the Independent Scientific Advisory Committee at the Medicines and Healthcare products Regulatory Agency. In addition, site‐specific approval to contact the GP practices was obtained for each of 132 primary care trusts across the United Kingdom, as described previously.^39^ Written informed consent was obtained from all study subjects or their guardians. The UKMYONET study was approved by the North West Research Multi‐Centre Research Ethics Committee (98/8/86), and all participants gave written informed consent.

Multicenter ethics approval was obtained from the South East Research Ethics Committee for the SEARCH study and from the local ethics committees covering each of the 69 UK hospitals involved in the Heart Protection Study.

The recruitment of cerivastatin case subjects was approved by the University of Washington Institutional Review Board, and the use of the Heart and Vascular Health study subjects was approved by the Group Health Subjects Review Committee.

### DNA extraction and genotyping

#### Case‐control “discovery” cohort

For the CPRD recruits (cases and controls), genomic DNA was extracted from 5 mL whole blood or 2 mL saliva (collected using the Oragene DNA Sampling kit; DNAGenotek, Ontario, Canada) using the Chemagic Magnetic Module (MSM) 1 system, as per the manufacturer's protocol (Chemagen Biopolymer‐Technologie AG, Baesweiler, Germany).

At the time of analysis, DNA samples from a total of 135 myopathy cases from the discovery cohort were available. At least 1.5 μg DNA from myopathy cases was genotyped for a total of 982,958 SNPs by ARK‐Genomics, University of Edinburgh (Edinburgh, UK) using the Illumina OmniExpress Exome version 1.0 Bead Chip array according to the manufacturers protocol (Illumina, San Diego, CA).

#### Discovery case‐control study‐population controls

Population control genotype data for the initial discovery case‐control GWAS was obtained from the Wellcome Trust Case‐Control Consortium 2 (WTCCC2) cohort of 2,501 individuals from the UK Blood Service.

#### Replication study

All 585 CPRD statin‐tolerant controls and 19 patients with myopathy from the EUDRAGENE cohort were genotyped for statistically significant association signals identified in the case‐control discovery GWAS (*P* < 5 × 10^−5^) using either the Agena MassArray iPLEX platform (Agena Biosciences, San Diego, CA) or TaqMan real‐time polymerase chain reaction SNP genotyping assay (Life Technologies, Paisley, UK), according to the manufacturer's protocols.

### Genotyping QC and imputation

#### Case‐control “discovery” cohort

Cases identified via CPRD recruitment were excluded if they failed to meet the following criteria: (i) gender as determined by the “Sex Check” function within PLINK^40^ differed from that reported in the clinical data; (ii) genotype call‐rate < 90%; and (iii) principle component analysis (using SNPRelate^41^ in R version 3.01) demonstrated that the individual did not cluster with the HapMap CEU (Utah residents with European ancestry) population (**Figure S1**).

SNPs, in both the discovery and replication cohorts, were excluded if (i) minor allele frequency was < 0.01, (ii) Hardy–Weinberg Equilibrium *P* value was < 0.0001, and (iii) the genotype success rate was < 95%. All QC analysis was undertaken using PLINK version 1.07^40^ unless otherwise stated.

For the purpose of the discovery case‐control study, the CPRD case genotype dataset was merged with the WTCCC dataset prior to SNP phasing using SHAPEIT^42^ and imputation using IMPUTE2^43^, ^44^ was undertaken using the 1000 Genomes Project phase III reference panel.

#### The “simvastatin” validation case‐control study

Genotype imputation was undertaken using minimac^45^ with the 1000 Genomes European reference panel. Data for the association signal SNPs were provided for the validation and meta‐analysis.

#### The “cerivastatin” validation case‐control study

Samples were excluded from analysis for gender mismatch or call rate < 95%. The following variant exclusions were applied to obtain a cleaned set of variants for imputation: call rate < 97%, Hardy–Weinberg Equilibrium *P* < 10^−5^, >2 duplicate errors or Mendelian inconsistencies (for reference Centre d'Etude du Polymophisme Humain (CEPH) trios), heterozygote frequency = 0. MaCH^45^ was used to pre‐phase the genotypes. The phased genotypes were imputed into a reference panel of 1,092 individuals of multiple ethnicities from the phase I (version 3) haplotypes of 1000 Genomes Project using minimac.^45^ Genotyping and SNP calling were performed using the Illumina 370CNV Bead Chip, as previously described.^7^ Data for the association signal SNPs were provided for the validation and meta‐analysis.

### Statistical analysis

The study design and statistical analysis are summarized in **Figure** 3. In the discovery phase, cases passing genotype QC (*n* = 128) recruited via CPRD were compared with WTCCC2 controls (*n* = 2,501) using a logistic regression analysis undertaken in SNPTest^46^ and adjusting for the first two principle components as covariates. All SNPs giving a *P* value for association of < 5 × 10^−5^ were genotyped in the statin‐tolerant cohort (*n* = 585) and EUDRAGENE cohort (*n* = 19), which together formed the replication cohort.

A univariate analysis of nongenetic covariates (χ^2^ for categorical outcomes and Student's *t*‐test for continuous variables) was undertaken (**Table** 1) using SPSS version 17.0. Variables demonstrating a *P* value < 0.10 between the discovery cohort cases and tolerant controls were carried forward and adjusted for in the SNP association analyses (**Table S1**). Logistic regression analysis of the candidate SNPs in the cases (discovery and replication) and statin‐tolerant controls was undertaken using SNPTest. Meta‐analysis of the combined discovery and replication cohorts along with the two validation studies was undertaken using a fixed‐effects model with inverse‐variant effect size weighting in GWAMA.^47^ Forest plots were prepared using the “forestplot” function in R.

## Funding

This work was funded by a grant from the e‐Health Initiative funded jointly by the Medical Research Council (reference: MC_qA137929), Wellcome Trust, Engineering and Physical Sciences Research Council, and Economic and Social Research Council, and HL078888. M.P. is an National Institute for Health Research (NIHR) Senior Investigator. M.P. also thanks the Medical Research Council Centre for Drug Safety Science and NIHR Collaborations for Leadership in Applied Health Research and Care North West Coast for infrastructure support. Collaborators of UKMYONET are acknowledged in ref. 36. This report includes independent research supported by the NIHR Biomedical Research Centre Funding Scheme. The views expressed in this publication are those of the authors and not necessarily those of the National Health Service (NHS), the NIHR, or the Department of Health.

This research was supported by the NIHR Biomedical Research Centre at Guy's and St Thomas’ NHS Foundation Trust and King's College London. The views expressed are those of the author(s) and not necessarily those of the NHS, the NIHR, or the Department of Health.

The EUDRAGENE project was supported with a concerted action grant from the European Commission 5th Framework QLRI‐CT‐2002‐02757, by the Serious Adverse Events Consortium (SAEC), and the NIHR Biomedical Research Centre at Guy's and St Thomas’ NHS Foundation Trust and King's College London.

## Conflict of Interest

B.M.P. serves on the Steering Committee of the Yale Open Data Access Project funded by Johnson & Johnson. No other author declares any conflict of interest.

## Author Contributions

D.F.C., B.F., A.L.J., B.M.P., and M.P. wrote the manuscript. M.P., T.vS., and B.M.P. designed research. D.F.C., E.Z., H.C., S.R.H., J.C.B., J.A.B., J.F., B.M.P., M.M., M.L.‐M., A.C., A.A., T.vS., and M.P. performed research. D.F.C, B.F., A.L.J., A.A., T.vS., and M.P. analyzed data.

## Supporting information


**Supplementary Material:** Tables and Figures.
