# Supplementary material for: Genomewide Association Study of Statin‐Induced Myopathy in Patients Recruited Using the UK Clinical Practice Research Datalink
Source: Clin Pharmacol Ther. 2019 Jul 31;106(6):1353–61. doi: 10.1002/cpt.1557 (PMC6896237; doi:10.1002/cpt.1557)
Supplement: Supplementary file 1 — Supplementary Material: Tables and Figures. [file CPT-106-1353-s001.docx]

**SUPPLEMENTARY DATA**

**Supplementary Table 1**. Discovery cohort case/control comparison of non-genetic clinical variables. All comparison analyses were undertaken using a chi-squared test except those variables marked * where an independent samples t-test was applied. # indicates missing data (29 tolerant, 6 myopathy). Values marked in **bold** text indicate p<0.1 where variables were carried forward for inclusion in the binary logistic regression model.

| **Variable** | **Controls**  **(n=585)** | **Myopathy**  **(n=128)** | **p-value** | **Severe Myopathy (n=32)** | **p-value** |
| --- | --- | --- | --- | --- | --- |
| **Statin at Index** |  |  |  |  |  |
| Simvastatin | 342 (58%) | 85 (66%) | 0.542 | 22 (69%) | 0.510 |
| Atorvastatin | 175 (30%) | 28 (22%) |  | 5 (16%) |  |
| Rosuvastatin | 29 (5%) | 7 (6%) |  | 3 (9%) |  |
| Fluvastatin | 11 (2%) | 2 (2%) |  | 1 (3%) |  |
| Pravastatin | 26 (4%) | 6 (4%) |  | 1 (3%) |  |
| Cerivastatin | 2 (<1%) | 0 (0%) |  | 0 (0%0 |  |
| **Mean Daily Dose (mg/day) (SD)*** | 30.8 (±15.9) | 32.3 (±19.4) | 0.413 | 44.7 (±24.2) | **0.003** |
| **Mean age (years) (SD)*** | 69.7 (±9.3) | 66.3 (±10.4) | **<0.001** | 66.8 (±10.1) | **0.093** |
| **Gender** | 64% M | 73% M | **0.040** | 56% M | 0.453 |
| **Mean BMI*** | 28.9 (±5.1) | 29.8 (±4.9) | **0.070** | 30.4 (±5.1) | 0.133 |
| **Smoking Status#** |  |  |  |  |  |
| Non-smoker | 236 (42%) | 50 (41%) | 0.487 | 18 (60%) | 0.139 |
| Ex-smoker | 237 (43%) | 58 (48%) |  | 10 (33%) |  |
| Smoker | 83 (15%) | 14 (11%) |  | 2 (7%) |  |
| **Co-medications in 6mths prior to index** | |  |  |  |  |
| Antihypertensive | 477 (82%) | 95 (74%) | **0.066** | 25 (78%) | 0.641 |
| CYP3A4 inhibitor^1^ | 72 (12%) | 18 (14%) | 0.560 | 4 (13%) | 1.000 |
| Known statin interactor (non-CYP3A4 substrate)^2^ | 51 (9%) | 10 (8%) | 0.862 | 4 (13%) | 0.516 |
| Oral corticosteroid | 23 (4%) | 3 (2%) | 0.601 | 1 (3%) | 1.000 |
| **Occurrence in previous 6mths or 2wks after index** | | |  |  |  |
| Cramps | 5 (<1%) | 8 (6%) | **<0.001** | 5 (16%) | **<0.001** |
| Myocardial Infarction | 5 (<1%) | 1 (<1%) | 1.000 | 1 (3%) | 0.274 |
| Renal failure | 16 (3%) | 3 (2%) | 1.000 | 2 (6%) | 0.237 |
| Trauma | 1 (<1%) | 1 (<1%) | 0.326 | 0 (0%) | 1.000 |
| **Previous history (any time prior to index)** | |  |  |  |  |
| Type 2 diabetes | 154 (26%) | 42 (33%) | 0.127 | 10 (31%) | 0.539 |
| Alcohol dependence | 26 (4%) | 7 (5%) | 0.642 | 1 (3%) | 1.000 |
| Asthma | 75 (13%) | 17 (13%) | 0.885 | 1 (3%) | 0.1612 |
| Atrial fibrillation | 61 (10%) | 12 (9%) | 0.872 | 5 (16%) | 0.373 |
| Chronic Obstructive Pulmonary Disease | 41(7%) | 7 (5%) | 0.697 | 0 (0%) | 0.260 |
| Hypertension | 381 (65%) | 63 (49%) | **0.001** | 19 (59%) | 0.569 |
| Hyperthyroidism | 10 (2%) | 3 (2%) | 0.713 | 0 (0%) | 1.000 |
| Hypothyroidism | 47 (8%) | 12 (9%) | 0.597 | 5 (16%) | 0.178 |

^1^CYP3A4 interacting co-medications were amiodarone, cyclosporine, azole antifungals, macrolide antibiotics, protease inhibitors, calcium channel blockers (see supplementary table 2). ^2^Non-CYP3A4 interacting co-medications recorded were Fenofibrate, Gemfibrozil, Digoxin, Warfarin, and Nicotinic Acid

**Supplementary Table 2.** Co-medications classified as CYP3A4 interactors for the purpose of this study

| **Class** | **Drug** |
| --- | --- |
| Azole anti-fungals | Clotrimazole |
|  | Fluconazole |
|  | Itraconazole |
|  | Voriconazole |
| Antibiotics | Amoxicillin |
|  | Azithromycin |
|  | Clarithromycin |
|  | Erythromycin |
|  | Telithromycin |
|  | Metronidazole |
| Calcium Channel Blockers | Diltiazem |
|  | Mibefradil |
|  | Verapamil |
| Protease Inhibitors | Atazanavir |
|  | Darunavir |
|  | Indinavir |
|  | Liponavir |
|  | Nelfinavir |
|  | Ritonavir |
|  | Saquinavir |
| Others | Amiodarone |
|  | Cyclosporine |
|  | Imatinib |
|  | Nefazodone Lansoprazole |

**Supplementary Table 3.** Summary association statistics for the 12 SNP signals identified from the discovery cohort analysis (“all myopathy” and “severe myopathy” phenotypes) limited to simvastatin or atorvastatin only all myopathy. n/a denotes analyses where summary statistics are not available due to lack of minor allele carriage in the case group.

|  |  | **SIMVASTATIN ONLY** | | **ATORVASTATIN ONLY** | |
| --- | --- | --- | --- | --- | --- |
|  | **rs** | **OR (CI)** | **p** | **OR (CI)** | **p** |
| **All myopathy** | **rs36121096** | n/a | n/a | n/a | n/a |
|  | **rs55902659** | 0.45 (0.28-0.72) | 0.0002 | 0.0364 | 0.46 (0.21-1.02) |
|  | **rs17359612** | 2.58 (1.64-4.05) | 0.0002 | 0.0095 | 2.94 (1.41-6.14) |
|  | **rs79860430** | 2.43 (1.51-3.93) | 0.0008 | 0.0261 | 2.67 (1.21-5.88) |
|  | **rs77855582** | 2.81 (1.34-5.89) | 0.0171 | n/a | n/a |
|  |  |  | |  |  |
| **Severe Myopathy** | **rs73089338** | 2.15 (1.42-3.25) | 0.0003 | 0.6417 | 0.80 (0.29-2.25) |
|  | **rs504365** | 0.75 (0.53-1.06) | 0.0986 | 0.6365 | 1.16 (0.67-2.02) |
|  | **rs2247256** | 0.69 (0.48-0.99) | 0.0380 | 0.5493 | 0.85 (0.47-1.53) |
|  | **rs117576073** | n/a | n/a | n/a | n/a |
|  | **rs4149056** | 2.09 (1.48-2.96) | 0.0001 | 0.2133 | 1.49 (0.79-2.84) |
|  | **rs4149000** | 2.22 (1.55-3.17) | 0.0001 | 0.1695 | 1.61 (0.83-3.12) |
|  | **rs28447350** | 1.25 (0.87-1.80) | 0.2284 | 0.0299 | 1.94 (1.10-3.42) |

**
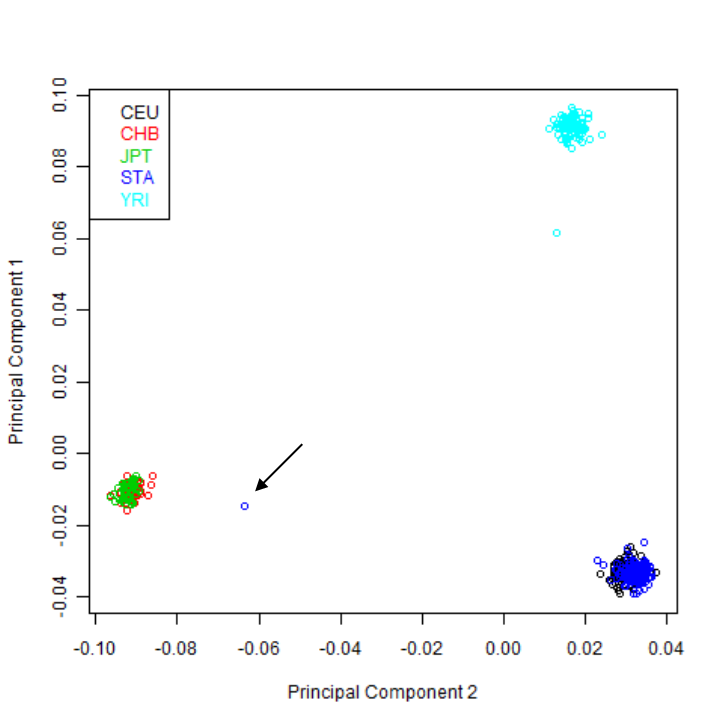
**

**Supplementary Figure 1.** Principal component analysis (PCA) of the statin myopathy discovery case cohort. PC1 is plotted on the Y-axis and PC2 on the x-axis. Statin myopathy cases (STA) are marked blue. The four HapMap populations CEU (Utah residents of European ancestry), CHB (Han Chinese), JPT, Japanese and YRI (Yoruban for Nigeria) are indicated by the key. The black arrow indicates an excluded outlier individual**.**

**Supplementary Figure 2.** Zoom plot of the *SLCO1B1* and *SLCO1A2* loci for the discovery cohort vs WTCCC genome-wide logistic regression analysis for A) all myopathy (n=128) and B) Severe myopathy (n=32).


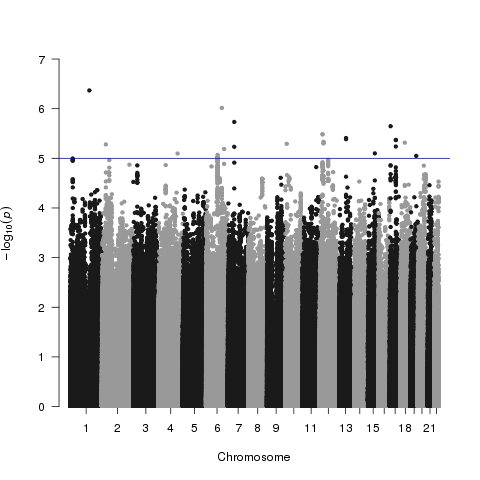


**Supplementary Figure 3**. Manhattan plot of genome-wide association analysis of simvastatin-induced myopathy. The data represents logistic regression derived log p-values (y-axis) of SNPs for the discovery case-control analysis of the “all myopathy” phenotype (n=85) with the WTCCC2 population controls (n=2,501). X-axis is the position of the SNP with the chromosome indicated. The blue line represents a notional significance threshold (p=5x10^-5^) and the red line.

**Supplementary Table 4.** Summary association statistics for the top 100 SNPs (by p-value) identified from the discovery cohort analysis limited to simvastatin only “all myopathy”. Where multiple SNPs are present for in locus signal, only the SNP with the lowest p-value is reported.

| **ID** | **Chr** | **Pos (hg 38)** | **Gene** | **OR** | **p-value** |
| --- | --- | --- | --- | --- | --- |
| rs190206717 | 1 | 80480922 | Intergenic | 43.9 (4.84-398.9) | 8.86E-06 |
| rs77051277 | 1 | 150167075 | *RP11-458I7.4* | 50.9 (10.6-243.6) | 4.29E-07 |
| rs62131018 | 2 | 29828498 | *ALK* | 1.99 (1.46-2.71) | 5.24E-06 |
| rs12508927 | 4 | 4603786 | *RP11-323F5.2* | 5.02 (2.29-11.0) | 6.05E-06 |
| rs140854723 | 4 | 5634585 | *EVC2* | 66.1 (10.6-414.0) | 1.15E-06 |
| rs116168042 | 6 | 11754825 | *ADTRP* | 2.76 (1.71-4.46) | 8.71E-06 |
| rs6454721 | 6 | 88769294 | *RNGTT* | 0.25 (0.12-0.54) | 8.55E-06 |
| rs10872257 | 6 | 122969896 | Intergenic | 0.46 (0.33-0.64) | 9.66E-07 |
| rs11155111 | 6 | 140380286 | Intergenic | 2.03 (1.49-2.75) | 6.48E-06 |
| rs7779564 | 7 | 48686597 | Intergenic | 3.37 (1.82-6.22) | 1.85E-06 |
| rs117119573 | 8 | 11028343 | *XKR6* | 9.07 (1.96-41.93) | 8.47E-06 |
| rs149657468 | 10 | 9555497 | Intergenic | 9.31 (1.97-43.9) | 9.58E-06 |
| rs10795948 | 10 | 12308135 | 3' of Metazoa_SRP | 0.47 (0.34-0.65) | 5.11E-06 |
| rs184787123 | 10 | 96947432 | *LCOR* | 15.8 (4.64-53.9) | 5.91E-06 |
| rs61865606 | 10 | 132440647 | *RP11-432J24.3* | 0.39 (0.21-0.72) | 2.52E-07 |
| rs11519272 | 12 | 21209458 | *SLCO1B1* | 2.37 (1.68-3.36) | 3.28E-06 |
| rs187058151 | 12 | 28675079 | Intergenic | 1.94 (1.40-2.68) | 4.63E-06 |
| rs145427387 | 13 | 70725682 | Intergenic | 2.33 (1.66-3.29) | 3.90E-06 |
| rs56027559 | 15 | 73858742 | *RP11-24D15.1* | 2.77 (1.86-4.12) | 7.94E-06 |
| rs138591431 | 15 | 75697275 | *CSPG4* | 3.92 (2.22-6.93) | 9.14E-06 |
| rs148352615 | 15 | 75740948 | *RP11-24M17.4* | 3.94 (2.23-6.97) | 7.14E-06 |
| rs190668351 | 16 | 49412462 | 3' of *C16orf78* | 22.3 (5.27-94.1) | 7.04E-06 |
| rs149208014 | 16 | 77648576 | Intergenic | 35.7 (4.06-314.6) | 6.72E-06 |
| rs333114 | 17 | 4495991 | 3' of *AC118754.4* | 3.22 (1.72-6.05) | 2.25E-06 |
| rs79980568 | 17 | 47671302 | *KPNB1* | 3.21 (2.02-5.08) | 5.78E-06 |
| rs72648866 | 17 | 47735928 | *TBX21* | 3.37 (2.13-5.34) | 4.25E-06 |
| rs2861137 | 18 | 37141725 | *KIAA1328* | >0.01(>0.001-1092) | 4.85E-06 |
| rs77180504 | 19 | 9623491 | *ZNF561* | 14.9 (3.99-55.6) | 5.55E-06 |
| rs41558212 | 19 | 45414987 | *ERCC1* | 3.18 (2.01-5.05) | 8.92E-06 |


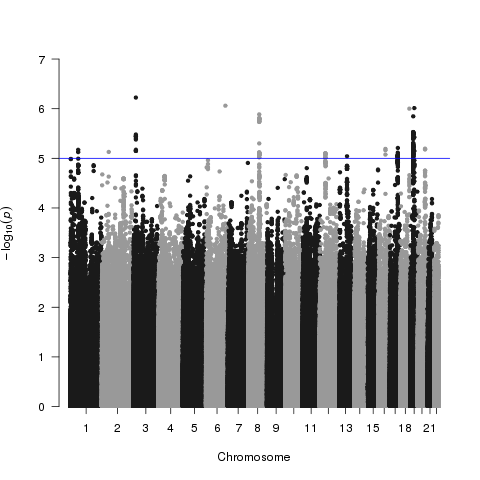


**Supplementary Figure 4**. Manhattan plot of genome-wide association analysis of atorvastatin-induced myopathy. The data represents logistic regression derived log p-values (y-axis) of SNPs for the discovery case-control analysis of the “all myopathy” phenotype (n=28) with the WTCCC2 population controls (n=2,501). X-axis is the position of the SNP with the chromosome indicated. The blue line represents a notional significance threshold (p=5x10^-5^).

**Supplementary Table 5.** Summary association statistics for the top 100 SNPs (by p-value) identified from the discovery cohort analysis limited to atorvastatin only “all myopathy”. Where multiple SNPs are present for in locus signal, only the SNP with the lowest p-value is reported.

| **ID** | **chr** | **Pos (hg 38)** | **Gene** | **OR** | **p-value** |
| --- | --- | --- | --- | --- | --- |
| rs9831928 | 3 | 21224048 | Intergenic | 0.21(0.09-0.46) | 3.31E-06 |
| rs4256319 | 5 | 7538189 | *ADCY2* | 0.02(<0.01-0.12) | 3.11E-06 |
| rs6925743 | 6 | 1.5E+08 | *PPP1R14C* | 0.19 (0.08-0.46) | 8.72E-07 |
| rs11780883 | 8 | 83834281 | Intergenic | 0.22 (0.11-0.44) | 1.30E-06 |
| rs10871700 | 18 | 72302261 | Intergenic | 0.30 (0.17-0.50) | 9.99E-07 |
| rs28420503 | 19 | 22133911 | *ZNF257* | 4.79 (2.17-10.56) | 3.83E-06 |
| rs34312380 | 19 | 22207269 | *ZNF676* | 0.21 (0.09-0.46) | 2.98E-06 |
| rs33428 | 19 | 30446936 | *ZNF536* | 0.26 (0.15-0.45) | 9.73E-07 |
| rs150707400 | 19 | 34352219 | *KIAA0355* | 64.6 (8.20-508.8) | 4.71E-06 |
